# Breakage and displacement of the high-speed hand-piece bur during impacted mandibular third molar extraction: three cases

**DOI:** 10.1186/s12903-022-02253-8

**Published:** 2022-06-06

**Authors:** Kuncai Li, Bingqing Xie, Junliang Chen, Yun He

**Affiliations:** 1 Oral and Maxillofacial Reconstruction and Regeneration Laboratory, Southwest Medical University, Luzhou, 646000 China; 2 Department of Oral and Maxillofacial Surgery, The Affiliated Stomatology Hospital of Southwest Medical University, Luzhou, China

**Keywords:** High-speed hand-piece, Bur segment, Mandibular third molar, Tooth extraction, Complication, Case report

## Abstract

**Background:**

The high-speed hand-piece bur is one of the methods to perform tooth sectioning during the minimally traumatic extraction of impacted mandibular third molars. During tooth sectioning, the breakage of the bur might take place when it is improperly used. Three cases of the breakage and displacement of a high-speed hand-piece bur during extraction are reported, aiming to remind dental surgeons of this complication.

**Case presentation:**

The bur fragment in case 1 was embedded in the mandibular bone under the previously removed crown of tooth 48 and distal to tooth 47. The bur fragment in case 2 was embedded in the lingual edge of the socket and partly beneath the mucosa on the lingual side. The position of the bur fragment in case 3 was similar to that of case 1 but was completely embedded in the spongious bone. The three cases were performed by first-year residents, and all of the bur fragments were successfully removed by attending doctors after accurately locating them by radiological examination.

**Conclusions:**

In order to avoid breakage of the high-speed hand-piece bur, the number of uses of the bur should be monitored and the integrity and state of the bur should be carefully checked. Moreover, light pressure with little lateral force should be used during tooth sectioning. If bur breakage and displacement occur, the retrieval protocol should be determined based on the imaging findings and conducted as soon as possible to avoid serious consequences.

## Background

Surgical removal of impacted mandibular third molars is one of the most common surgical procedures performed in dentoalveolar surgery. Nowadays, minimally traumatic tooth extraction is widely applied, since it improves the clinical outcome, including reducing healing time, discomfort and inflammation [1, 2]. Furthermore, a contra-angle high-speed hand-piece bur made of steel with a tungsten carbide or diamond coating is one of the methods used for tooth sectioning during minimally traumatic extraction of impacted mandibular third molars [3–5]. During tooth sectioning, more than one cut is frequently necessary in the impacted tooth, and the breakage of the high-speed hand-piece bur might take place during extraction of an impacted mandibular third molar when the instrument is not properly selected and the bur improperly used [5, 6]. It obliges to search for the broken fragment and remove it to avoid possible infection or to prevent complications due to swallowing or aspiration of the fragment [7]. Here, three cases of the breakage and displacement of a high-speed hand-piece bur during mandibular third molar extraction are reported, aiming to remind dental surgeons of this uncommon complication in dentoalveolar surgery.

## Case presentation

**Fig. 1:**
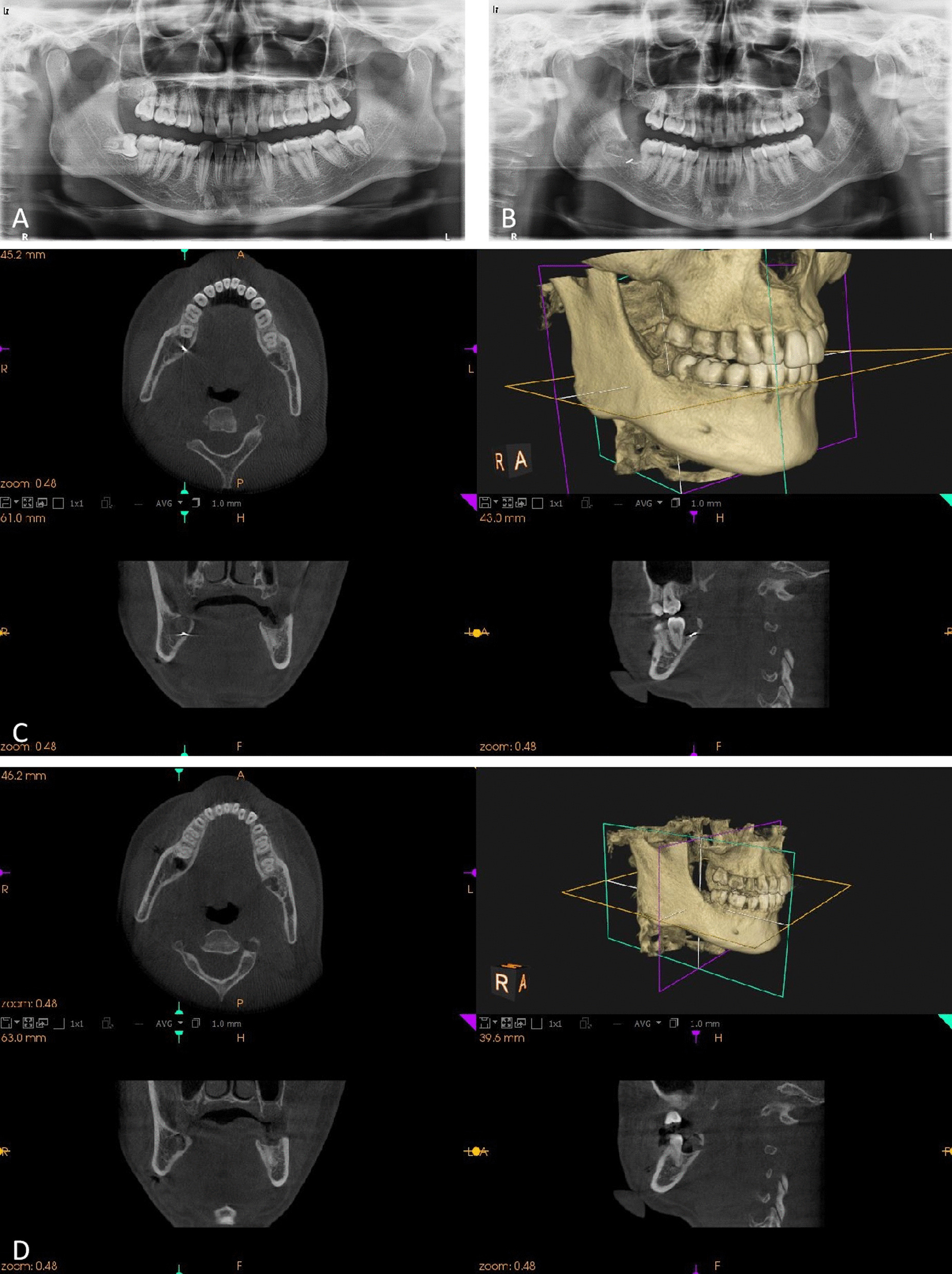
*Case 1* **A** Preoperative panoramic radiograph. **B** Panoramic radiograph showing the bur fragment in the socket of the right mandibular third molar. **C** CBCT showing the 3D position of the bur fragment. **D** CBCT confirmed the removal of the bur fragment

**Fig. 2:**
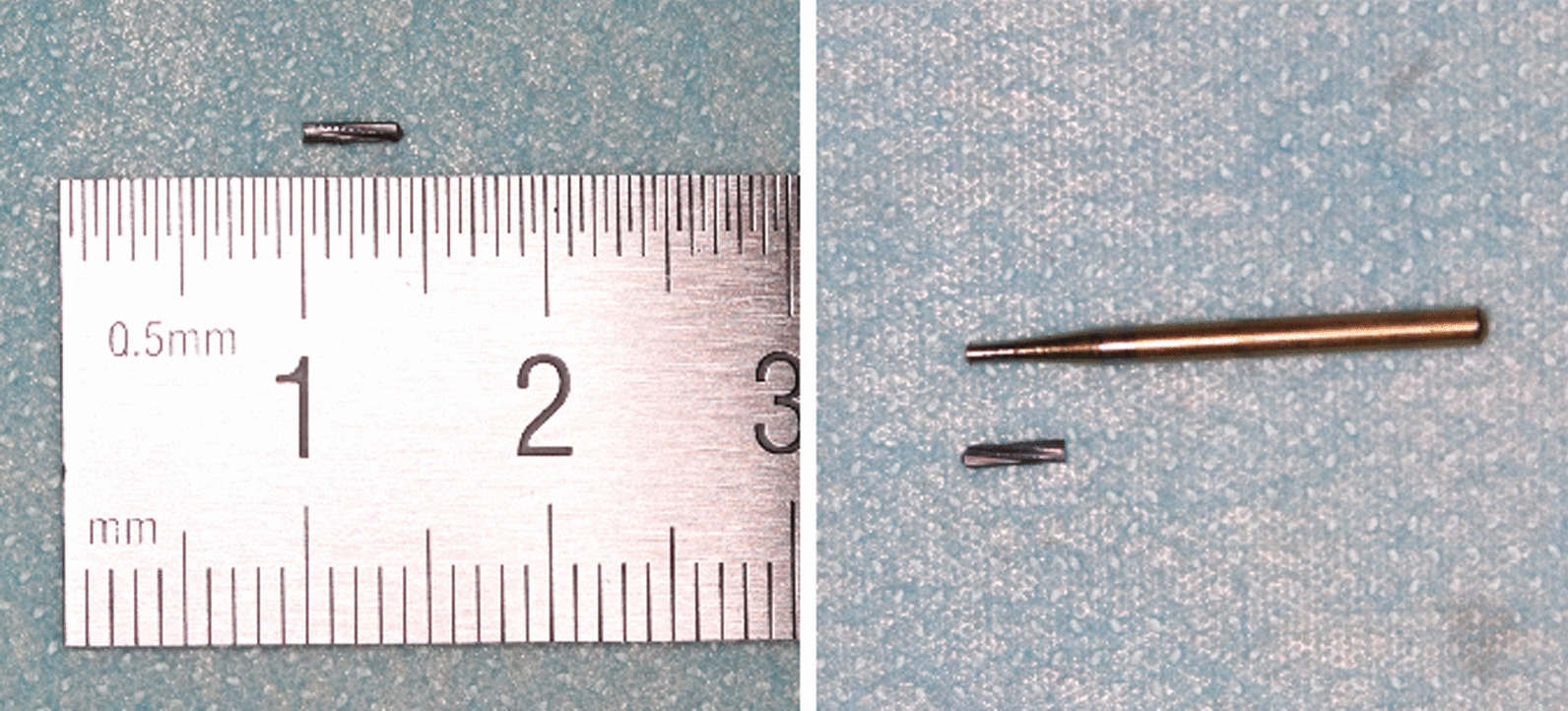
The bur fragment

The first patient was a 24-year-old male who was undergoing extraction of a right impacted mandibular third molar (tooth 48). According to the classification of Pell and Gregory [8] and Winter [9], it was a class II (the space between the distal portion of the second molar and the ramus of the mandible is less than the mesiodistal diameter of the crown of the third molar), position B (the highest portion of the impacted third molar is below the occlusal plane but above the cervical line of the second molar) horizontal (the long axis of the third molar is perpendicular to the long axis of the second molar) impacted tooth. During tooth sectioning, the surgical fissure bur broke accidentally and then displaced. It was difficult to find the bur fragment immediately because of poor visibility due to blood, saliva and the tooth. After extracting the tooth, a panoramic radiograph and cone beam computed tomography (CBCT) were performed to identify the size and exact position of the bur fragment. The bur fragment was found embedded in the mandibular bone under the previously removed crown of tooth 48 and distal to tooth 47 (Fig. 1). The excessive cutting depth was probably the reason why the bur became stuck in the spongious bone beneath the crown during sectioning of the crown and subsequently broke. A careful search was conducted in the socket accompanied by suctioning. After locating the bur fragment, it was removed by using dental tweezers (Fig. 2). CBCT was used to confirm the removal of the bur fragment at the patient’s request. The postoperative course was uneventful.

**Fig. 3:**
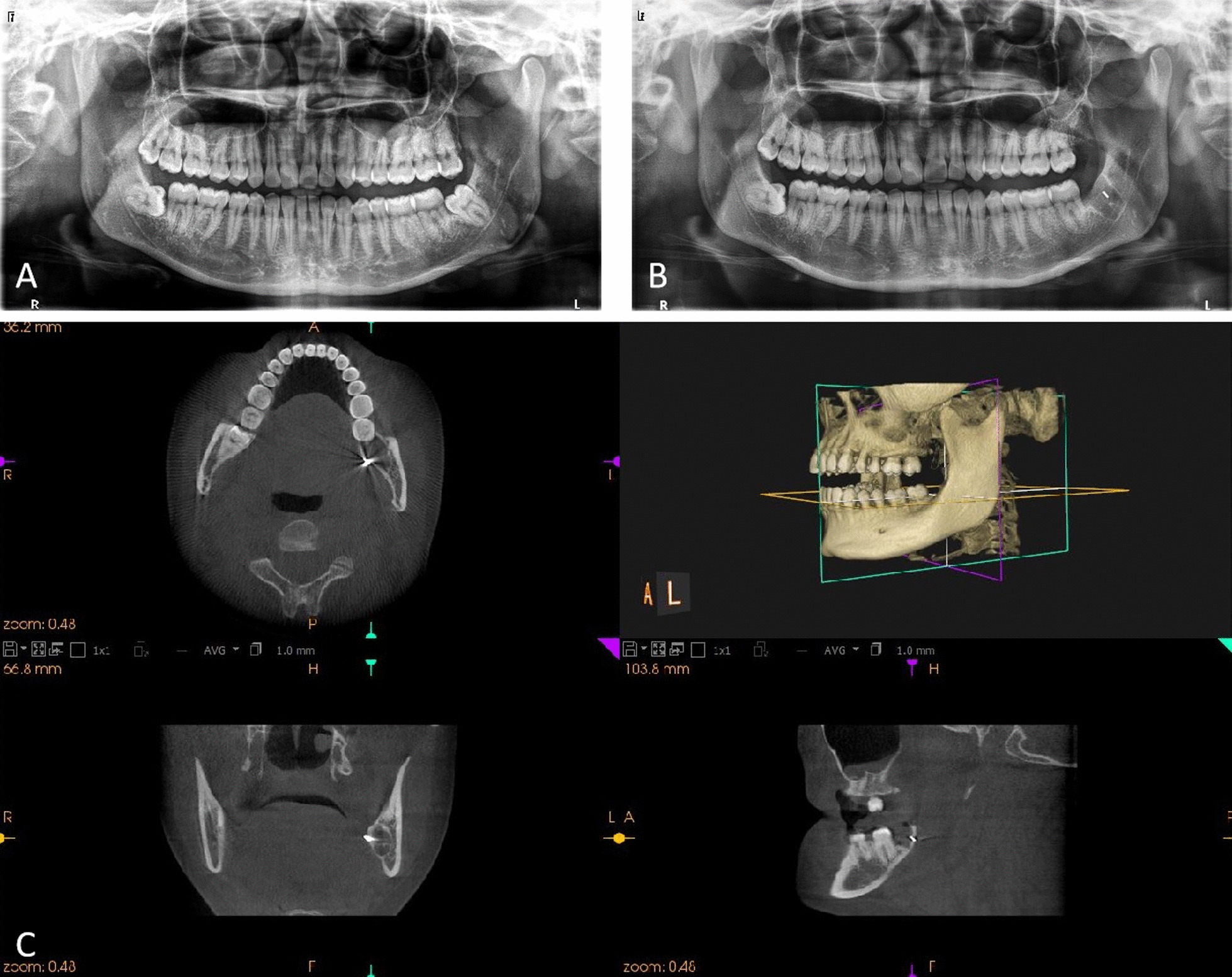
*Case 2* **A** Preoperative panoramic radiograph. **B** Panoramic radiograph showing the bur fragment in the socket of the left mandibular third molar. **C** CBCT showing the 3D position of the bur fragment

The second patient was a 30-year-old female who was undergoing extraction of a left impacted mandibular third molar (Fig. 3). This was a class II, position B mesioangular (the long axis of the tooth is inclined towards the second molar) impacted tooth. The bur fragment was found embedded in the lingual edge of the socket and partly beneath the mucosa of the lingual side. After locating the bur fragment in the socket, it was removed by using haemostatic forceps while compressing the submandibular region using the finger of the left hand.

**Fig. 4:**
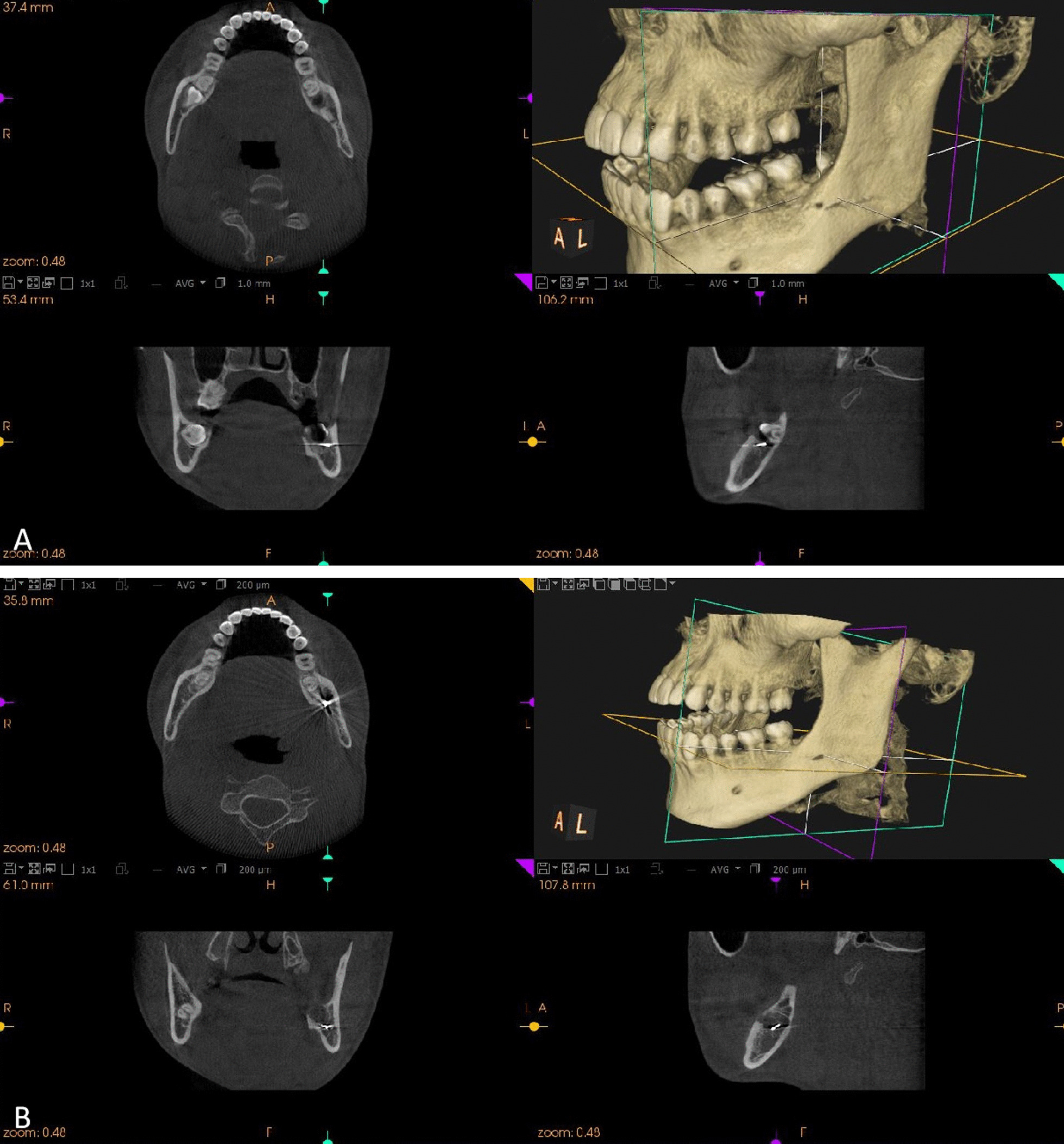
*Case 3* **A** The breakage of the bur during crown sectioning. CBCT immediately showed the 3D position of the bur fragment. **B** CBCT showing the 3D position of the bur fragment 5 days after tooth extraction

The third patient was a 25-year-old male with a left impacted mandibular third molar (Fig. 4). This was a class II, position B horizontal impacted tooth. The bur broke during crown sectioning, and the location of the bur fragment was similar to that in the first patient, except that it was fully embedded in the mandibular bone. Removal failed, even after the tooth had been extracted. Since the patient was too tired to maintain sufficient mouth opening for subsequent surgical treatment, the socket was sutured first. Then, the patient returned 5 days later and underwent CBCT, which found that the bur fragment was also embedded in the mandibular bone but had moved a little to the lingual side. The bur fragment was positioned close to the superior cortex of the inferior alveolar canal. Thereafter, the socket was thoroughly washed with saline solution under local anaesthesia in order to clearly locate the exact position of the bur fragment. The bur fragment was removed using a dental probe and haemostatic forceps.

## Discussion and conclusions

Despite efforts to perform tooth removal carefully, accidents may happen sometimes. Among the tooth extraction instruments, the dental elevator and high-speed bur have a relatively thin working tip. The breakage and migration of their working tip might take place, although this has rarely been reported. If the pieces of broken instrument are left in the body, symptoms such as swelling and pain associated with infection may follow [10]. da Silva Pierro et al. [11] reported a case of a paediatric dental elevator fracture during a primary mandibular right second molar extraction. In this case, the elevator’s broken edge was lodged in the lateral part of the distal root of the extracted primary molar. Matsuda et al. [12] reported two cases of migration of a high-speed dental hand-piece bur during mandibular third molar extraction, one with the iatrogenic foreign body migrating into the mandibular body that was subsequently removed under general anaesthesia and another case in which the iatrogenic foreign body migrated into the floor of the mouth and was removed under local anaesthesia. These two cases were not treated in the author’s department and did not report the process of bur breakage.

Tooth sectioning related to impacted tooth extraction is a daily procedure in oral and maxillofacial surgery. High-speed dental hand-piece burs have a high cutting efficiency and might be acquired in every dental clinic to become one of the minimally traumatic methods for tooth sectioning. However, dentists should always pay attention to the probability of overheating, subcutaneous emphysema and/or breakage [13, 14]. Surgical fissure burs and diamond burs are the two most frequently used for tooth sectioning by high-speed dental hand-piece during the extraction of impacted mandibular third molars. Compared to the diamond bur, the surgical fissure bur, which is made of tungsten carbide, has a much longer length (maximum 28 mm) and a relatively short (4–5 mm) and thin (around 1 mm) working area. These characteristics avoid damage to the neighbouring tooth and soft tissue. However, they make the bur more susceptible to breakage.

Between December 2018 and July 2020, there were three cases of breakage and displacement of a high-speed hand-piece bur in the Department of Oral and Maxillofacial Surgery at the Affiliated Stomatology Hospital of Southwest Medical University, as described above. The incidence rate was around 0.06%. All cases in this report involved breakage of a surgical fissure bur, with the same broken point. The burs used in all cases were the same model products from the same company. According to the product instructions, the bur is not disposable and can be reused after strict high-pressure steam sterilization. On the other hand, the three cases were performed by three first-year residents, and the removal of bur fragment was performed by attending doctors. Based on these three cases, the following measures should be taken to avoid breakage of a high-speed hand-piece bur.

First, the number of uses of the bur used should be monitored. Although the high-speed bur used in dental clinical practice may be subjected to fatigue from sterilization, it might also become worn and break after using it several times. It is advisable to retire the bur after more than 20 uses and to use reliable brands and products with good quality [15]. The integrity of the instrument should be checked before and after each surgical procedure. However, the number of uses of the bur is not recorded in most clinics. Normally, the bur is replaced as its cutting efficiency is decreased or its cutting edge is worn. The overuse of the bur might lead to its breakage. Second, adequate irrigation is important to keep the temperature of the bur and the surrounding tissue within an acceptable range and to remove debris for improved visibility [3]. This is also essential in order to avoid overheating of the bone and hand-piece. The typically accepted threshold temperature and “danger zone” for bone survival is 47 ℃ for longer than 1 min [16, 17]. For the three reported cases, the dental chair unit waterlines with aquae sterilisata was used for adequate irrigation during the sectioning. Third, light pressure with little lateral force should be used during the process of cutting, and the sectioning of the tooth crown should be restricted to the tooth rather than extending into the bone under the crown. Otherwise, the bur might become stuck in the tooth or the bone and break. Residents with relatively poor clinical experience might ignore this point. Therefore, the reasons for the bur breakage in the three cases might be due to excessive times of use and excessive cutting depth and pressure with lateral force.

If breakage and migration of the bur do occur, the position of the broken fragment should first be confirmed by imaging. In the three cases, a panoramic radiograph was first used to confirm the existence and overall information of the bur fragment, since the bur fragment might be sucked in by the saliva ejector. Moreover, the reason for using a panoramic radiograph was its cheaper price and less radiation compared to CBCT. On the other hand, CBCT might be the optimum imaging examination, since it provides a three-dimensional image. However, the accurate location of the segment might be impaired by metal artifacts [18]. Dentists must have good ability in spatial thinking when reading CBCT information. Moreover, a dental operating microscope or magnifying glass can be also used to locate the bur fragment and assist in the removal of the bur fragment. However, not all clinics are equipped with a dental operating microscope and magnifying glass; our department does not. Therefore, the combination of panoramic radiograph and CBCT was used to identify the size and exact position of the bur fragment in the three cases. Finally, in order to confirm the complete removal of the bur fragment, it has to be compared with the images and the remaining part of the bur after its removal. Furthermore, panoramic radiograph or CBCT could be used to confirm its removal. The first patient received CBCT to confirm the complete removal of the bur fragment due to the patient’s request. Besides, the periapical radiograph was not used in the three cases, since its insufficient image information and the patients’ discomfort in periapical radiographic examination of the mandibular third molar region [19].

Moreover, it is wise to remove the fragment as soon as possible in order to prevent it from migrating into a neighbouring space. The delayed removal of the fragment might carry the risk of infection, thrombosis, erosion into the carotid artery or one of its branches and interference with some cranial nerves [20]. The first and second patients in this report underwent removal of the fragment as soon as possible after tooth extraction. However, the third patient underwent removal 5 days after tooth extraction. The reason being that the patient was too tired to maintain sufficient mouth opening for good vision and subsequent surgical treatment, since the tooth extraction surgery had taken a relatively long time. Local swelling and pain also delayed the removal of the bur for 2 days after tooth extraction. The removal surgery was successfully performed 5 days after tooth extraction, when the swelling and pain was alleviated and mouth opening was sufficient. Another reason was that the fragment was completely and tightly lodged in the spongious bone according to CBCT. Thus, we assumed that it was not likely to move into the neighbouring tissue within a short space of time. However, the subsequent CBCT 5 days later indicated that the fragment had moved a little to the lingual side. Therefore, it might be better to remove the bur segment during or immediately after tooth extraction.

In conclusion, the following learning points about the prevention and treatment of bur breakage could be drawn. Firstly, the number of uses of the bur should be monitored, and it should be retired within 20 used during tooth sectioning related to impacted tooth extraction. The integrity and state of the bur should be carefully checked to avoid excessive wear and tear. Secondly, adequate irrigation during the operation is important and helps to reduce the temperature of the bur and its surrounding tissue, as well as prevent breakage of the bur to a certain extent [21]. Thirdly, light pressure with little lateral force should be used during the process of cutting, and the sectioning of the tooth crown should be restricted to the tooth. Finally, once the bur breakage and displacement occur, the specific location should be firstly confirmed by radiological examination. The retrieval protocol should be determined based on the imaging findings and conducted as soon as possible.

## Data Availability

The datasets used and/or analyses during the current study available from the corresponding author on reasonable request.
